# Identification problems of travelling snail species—new exotic introductions to tropical greenhouses in Gothenburg, Sweden (Gastropoda: Achatinellidae, Strobilopsidae, Helicarionidae)

**DOI:** 10.7717/peerj.11185

**Published:** 2021-04-06

**Authors:** Ira Richling, Ted von Proschwitz

**Affiliations:** 1 Staatliches Museum für Naturkunde Stuttgart, Stuttgart, Germany; 2 Gothenburg Natural History Museum, Gothenburg, Sweden; 3 Gothenburg Global Biodiversity Centre, University of Gothenburg, Gothenburg, Sweden

**Keywords:** Introduced species, Greenhouses, *Tornatellides* cf. *boeningi*, *Discostrobilops hubbardi*, *Ovachlamys fulgens*, Horticultural trade, Identification, Snails, Mollusca, COI

## Abstract

Three previously unreported species of tropical land snails were found in the greenhouses of the Gothenburg (Göteborg) Botanical Garden and the Public Science Center Universeum in Gothenburg. For *Tornatellides* cf. *boeningi* (Schmacker & Boettger, 1891) and *Ovachlamys fulgens* (*Gude, 1900*) this is the first observed occurrence in a European greenhouse, while *Discostrobilops hubbardi* (*Brown, 1861*) was first reported very recently in the Vienna Botanical garden. *Tornatellides* and *Discostrobilops* seem to be spread with orchid culture and trade. Identification of the *Tornatellides* species proved extremely difficult and a genetic sequence-based approach completely failed due to the unavailability of reference data. This was unexpected considering the importance of these introduced species in horticultural trade. A broader assessment of available sequence data for genetic identification based on COI or 16S for other snail species reported from horticultural facilities showed that such reference data in GenBank are still scarce and only for a limited number of species this approach would support identification.

## Introduction

Along with the ever increasing global trade more and more species are translocated. Studying the malacofauna in European greenhouses may provide a snapshot of newly travelling species in horticulture, the latter forming one important pathway for the introduction of alien snail species (Cowie & Robinson, 2003; Bergey et al., 2014). While a certain set of foreign species has long been established in greenhouses, e.g. certain species of Subulininae and Gastrodontoidea as well as a few slugs (e.g. Anderson, 2005), a whole new set of exotic species is currently arriving, among them *Sterkia antillensis* *Pilsbry, 1920*, *Pupisoma* cf. *macneilli* (*Clapp, 1918*), different species of Euconulidae and *Paropeas achatinaceum* (*L. Pfeiffer, 1846*) (Da Sois, 2015; Proschwitz, 2016; Reischütz et al., 2018; Horsák, Naggs & Backeljau, 2020).

Their proper identification often causes significant problems. This is especially true in land snails in greenhouses because introduced species often originate from tropical areas that are poorly studied, i.e. reliable revisions simply do not exist for major families, regions and especially for small-sized species. An additional difficulty is added by the fact that in most cases the origin is primarily unknown. Due to high levels of convergence in shell morphology in various families among representatives from different continents, the search for candidate species becomes even more complex. On the other hand, correct identification is not just an intellectual exercise, but may prove crucial to evaluate the invasive potential of the respective newcomer. Proper identification may also help to narrow down the origin of the species and thus provide important information on its ecological requirements.

Here we tested two different identification approaches (conchological and genetic) on a new alien found in a Swedish greenhouse, report on two other newcomers, and evaluate the feasibility of genetic identification of a set of snail species reported from the horticultural trade.

## Sampling location and methods

### Gothenburg (Göteborg) Botanical Garden

The Gothenburg Botanical Garden, Sweden, contains a complex of greenhouses (57°40′59″N, 11°57′08″ E) for the culture and show of several tropical plants. It is basically split into different sections according to moisture and temperature preferences of the respective plants, three of them especially dedicated to orchids: *Vanilla*- and adjacent Tropical house: high humidity, temperature >20 °C; *Cattleya*-house: temperature >15 °C (tropic/subtropic orchids); *Dendrobium*-house: temperature >12 °C (subtropic orchids). Details on the snails found are given in “results” under the respective species.

### Methods

Identification of the introduced species was based on morphological characters, which were sufficient for two of the three species, as well as on mitochondrial DNA sequence data for *Tornatellides*. For two specimens, total DNA was extracted from foot tissue using the DNeasy Tissue Kit (Qiagen, Hilden, Germany). DNA amplification of the barcoding fragment of cytochrome oxidase subunit I gene (hereafter COI) and 16S ribosomal RNA gene (16S) was done in a total volume of 25 µl using the standard primers LCO1490 and HCO2198 (Folmer et al., 1994) and 16Sar and 16Sbr (Palumbi et al., 1991) respectively, Taq polymerase FastGene Optima (Nippon Genetics) with an annealing temperature of 50 °C for COI and 52 °C for 16S with 35 cycles. Purification and sequencing was done by lgc genomics (Berlin, Germany), the chromatograms and alignments were checked manually in BioEdit (Hall, 1999).

For potential molecular identification of the *Tornatellides* species, available reference sequence data were searched in GenBank (Benson et al., 2017) by using the nucleotide BLAST^®^-tool as well as a search via taxonomy (genera of Tornatellidinae and Tornatellininae as represented in MolluscaBase (2019)) (Table 1). All sequences found were used for comparison via pair-wise or between group *p*-distance calculations respectively, performed with MEGA6 (Tamura et al., 2013).

**Table 1:** Sequences used in this study.

Taxon	Locality	GenBank-accession-number		Source
		COI	16S	
*Tornatellides* cf. *boeningi* (SMNS-ZI0144094)	Gothenburg	MW354671	MW354514	Present study
*Tornatellides boeningi*	Japan, Ogasawara Ids.: Hahajima	–	HQ829922–HQ829932 GQ905620–GQ905680	Wada, Kawakami & Chiba (2012)
			GU457351–GU457399	S. Wada, 2010, unpublished
*Tornatellides* sp.	Hawaiian Ids.: Molokai	MT519859	MT519878	Yeung et al. (2020)
*Tornatellides* sp.	Hawaiian Ids.: Oahu	AY148504	–	Holland & Hadfield (2004)
*Elasmias* sp.	Hawaiian Ids.: Maui	AY148499	–	B.S. Holland, 2002, unpublished
*Tornatellaria* sp.	Hawaiian Ids.: Maui	MT519858	–	Yeung et al. (2020)
*Elasmias luakahaense* Pilsbry & Cooke, 1915	Hawaiian Ids.: Oahu	AY148498	–	Holland & Hadfield (2004)
*Elasmias peaseanum* (*Garrett, 1884*)	Moorea	AY148503	–	Holland & Hadfield (2004)

In order to check the general feasibility of molecular identification we analysed the availability of genetic data for a greater set of snail species relevant in horticultural trade, thus for the focus of our study. We based our selection on the fairly comprehensive and representative list of species recorded in horticultural facilities on the Hawaiian Islands (Cowie et al., 2008) and excluded the Hawaiian native species. GenBank was searched for available sequence data for COI and 16S for all respective species by the taxonomy search tool in nucleotides. The search was specified for larger datasets with search terms “cytochrome” or/and “oxidase” for COI because the gene regions of the sequences in GenBank are not consistently labelled. Results were either visually checked for the correct gene region in the annotations or the sequences downloaded and checked in an alignment. A reverse search with the BLAST^®^-tool for sequence data of unidentified species with one of the identified sequences was not performed. Similarly, neither data quality nor taxon match were checked because of the impossibility for all taxa investigated.

For the sequence data at the genus level, generic assignment and species count followed the status in MolluscaBase (2020) as the more reliable source and not in GenBank which leads to differences, e.g. *Bradybaena* in GenBank includes 14 species of which only seven are treated in this genus by MolluscaBase. Sequence information of unidentified species was not considered.

## Results

### *Tornatellides* cf. *boeningi* (Schmacker & Boettger, 1891)

*Tornatellina boeningi* Schmacker & Boettger, 1891: 180–181, pl. 2, fig. 7: Tamsui, Nord-Formosa

*Tornatellina boeningi*—Yen, 1939: 73, pl. 6, figs. 25–26: “type locality: Takao, Formosa”; additional material from Shekko, Hongkong; lectotype SMF 42552 (fig. 26: “Takoa, S. Formosa”)

*Tornatellides boeningi*—Sasaki, 2008: 192, fig. 15B

*Tornatellides boeningi*—Wada, Kawakami & Chiba, 2012: Hahajima Island of the Ogasawara Islands: distribution by birds

Material investigated: Gothenburg (Göteborg) Botanical Garden, greenhouses: leg. J. Ennerfelt, 2016 (GNM 308512/1 spm.); leg. J. Roth & T. von Proschwitz, 3.3.2017 (*Vanilla*-house: GNM 308493/20 spms. + few observed; Tropical house: GNM 308480/1 spm.); Tropical house, leg. Ira Richling, 18.8.2017 (SMNS-ZI0100695/3 spms., SMNS-ZI0144090/1 spm., SMNS-ZI0144091/12 spms., SMNS-ZI0144094/21 spms. + 2 DNA extracts); leg. T. von Proschwitz, 15.2.2019 (*Vanilla*-house: GNM 308504/32 spms., *Cattleya*-house: GNM 308500/15 spms., *Dendrobium*-house: GNM 308509/2 spms.)

#### Morphological identification

**Figure 1:**
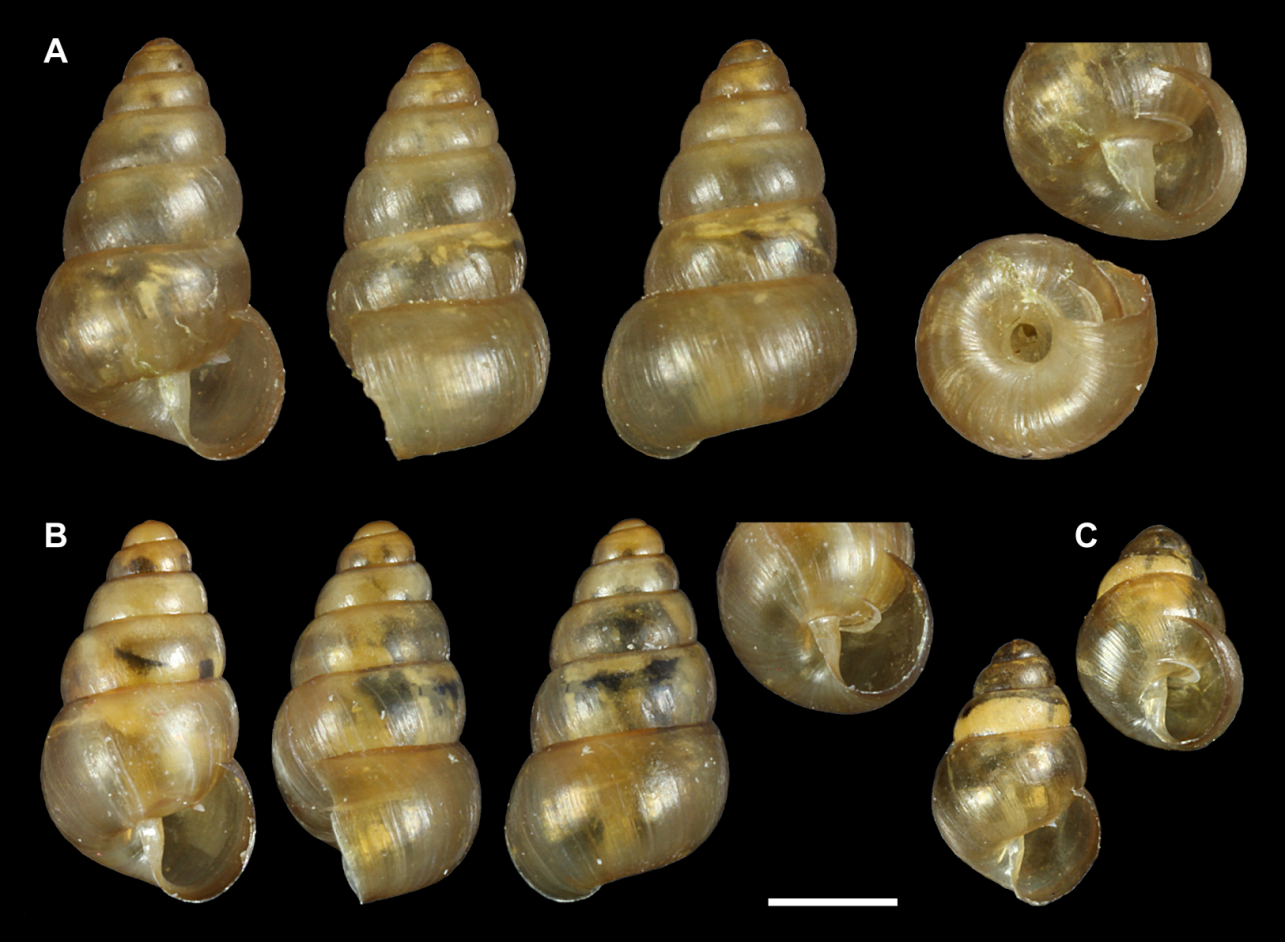
*Tornatellides* cf. *boeningi*, Gothenburg Botanical Garden. (A) Fully grown specimen, 3.3 mm height, GNM 308512, (B) nearly fully grown specimen, 3.0 mm height, SMNS-ZI0144090, (C) subadult specimen, 2.1 mm height, GNM 308493; scale bar = 1 mm (Photo credit: Ira Richling).

The greenhouse specimens are up to 3.3 mm high with up to 5.5 whorls, the whorls are well rounded with a moderately impressed suture. The umbilicus is small in juveniles to nearly fully grown adults, but significantly opens up in fully developed specimens. A sharp and slightly elevated parietal lamella is present throughout growth, and there is never a columellar lamella; the columella may be slightly curved (Fig. 1).

Identification of species of *Tornatellides* *Pilsbry, 1910*, especially of unknown origin, is difficult, because of the absence of a modern revision and the rather subtle conchological differences. The detailed anatomical studies for the whole family Achatinellidae by Cooke & Kondo (1961) primarily focused on classification and differentiation above species level. Nonetheless, they dissected ten different species of *Tornatellides* from five different “groups” (see below) and did not indicate any anatomical features enabling species identification. Furthermore, preliminary molecular studies so far presented only at conferences (e.g. Yeung et al., 2018) on Hawaiian representatives—the Hawaiian Islands being one major centre of diversification—even suggest a higher diversity and limitations in the conchological discrimination of the species. Given this situation, and although more than 100 years old, the revision of Pilsbry & Cooke (1914–1916) in the Manual of Conchology seems still the most reliable and comprehensive basic source of systematic information combined with the updates published by Cooke & Kondo (1961, especially p. 247).

Following the identification keys by Pilsbry & Cooke, we concluded that the greenhouse species could either belong to the “*simplex*-group” or possibly the “*perkinsi*-group”, the latter, however, being ruled out by the defining presence of clearly developed columellar lamellae in juveniles which our species is missing. The “*simplex*-group” of Pilsbry & Cooke (1914–1916: 195–215) includes representatives as geographically diverse as from the islands of Japan, Taiwan, Polynesia, New Zealand, Galapagos and Hawaii summing to 21 species and two subspecies. With corrections and additions made or summarized by Cooke & Kondo (1961) the now called “*oblongus*-group” (*T. simplex* (*Pease, 1865*) is regarded as a synonym of *T. oblongus* (*Anton, 1839*)) contains 22 species including 12 Hawaiian representatives.

Of those potential candidates the following seven non-Hawaiian species were excluded for the reasons given in parentheses: *T. inexpectatus* (*Pilsbry, 1901*) (species status questioned by Pilsbry & Cooke (1914–1916) and subsequently treated as a subspecies of *T. boeningi*, e.g. Takara, 1954), *T. tryoni* Pilsbry & Cooke, 1915 (different shape, less strongly developed parietal lamella), *T. perforatus* (*Liardet, 1876*) (regarded as *Lamellidea pusilla* (*Gould, 1847*) by Cooke & Kondo, 1961), *T. subperforatus* (*Suter, 1909*) (fewer whorls, more rapidly expanding, columellar lamella developed in juveniles), *T. inconspicuus* (Brazier, 1872) (regarded as belonging to *Tornatellinops* Pilsbry & Cooke, 1915 by Cooke & Kondo, 1961 and conchologically different: “columella twisted and entering spirally” as stated in the orginal description by Brazier, 1872 and confirmed with material from Lord Howe Island by Iredale, 1844: 308, pl. 18, fig. 7; currently synonymised with *Tornatellinops jacksonensis* (*Cox, 1864*) by Stanisic et al., 2018), *T. clarionensis* Dall, 1926 (p. 485, pl. 35, fig. 9: unusually large, whorls less convex, parietal lamella hardly developed), *T. mexicana* Dall, 1926 (p. 484–485, pl. 35, fig. 8: clearly distinct by two columellar lamellae), leaving *T. boeningi*, *T. oblongus* (*Anton, 1839*) and *T. chathamensis* (*Dall, 1892*) as possible identifications.

Among the Hawaiian species, the key in Pilsbry & Cooke (1914–1916: 202–203) leads to either *T. procerulus* (*Ancey, 1904*) or *T. kilauea* Pilsbry & Cooke, 1915 (our specimens’ diameter is about 58–59% of the height). However, it may be seriously questioned whether the first option (diameter less than 50% of the length) leading to *T. konaensis* *Cooke & Pilsbry, 1915*/*T. kahoolavensis* *Cooke & Pilsbry, 1915* is correct because measurements of the respective figures lead to ratios of 55% or 58% (as for *procerulus*). In any way, *T. konaensis* and *T. kahoolavensis* are ruled out by size or shape of the umbilicus. *T. procerulus* (different shape of the base, higher aperture and higher ultimate whorl) and *T. kilauea* (too broad and rapidly expanding) also do not match our species. In addition, all Hawaiian species appear to have a straighter outline or less convex whorls (see also figures in Severns (2011)).

From the three remaining non-Hawaiian candidates, the Galapagos species *T. chathamensis* was illustrated with its syntype and recent material by Miquel & Herrera (2014: 110–112, figs. 5–7). Considering size (taken from the image, 3.1 mm high) and number of whorls the syntype seems fully grown. Despite a certain similarity, compared to our larger specimen with the same number of whorls, it has a smaller umbilicus and mainly lacks the specific widening of the last whorl (compare Figs. 1A and 1B), so we regard it as different. In addition, Cooke & Kondo (1961) describe “one or two moderately to well-developed columellar folds in juvenile specimens” for material from Galapagos in the Bishop Museum collection, providing another argument against this identification.

A comparison with *T. oblongus* is complicated by the wide distribution of the species ranging throughout almost all suitable southeast Pacific islands from the Society Islands in the west, reaching Pitcairn in the southeast and up to the Marquesas in the north, going along with a considerable variation in shell shape and the development of the parietal lamella (Cooke & Kondo, 1961). As a result, we regard it impossible to distinguish our species morphologically from *T. oblongus*.

**Figure 2:**
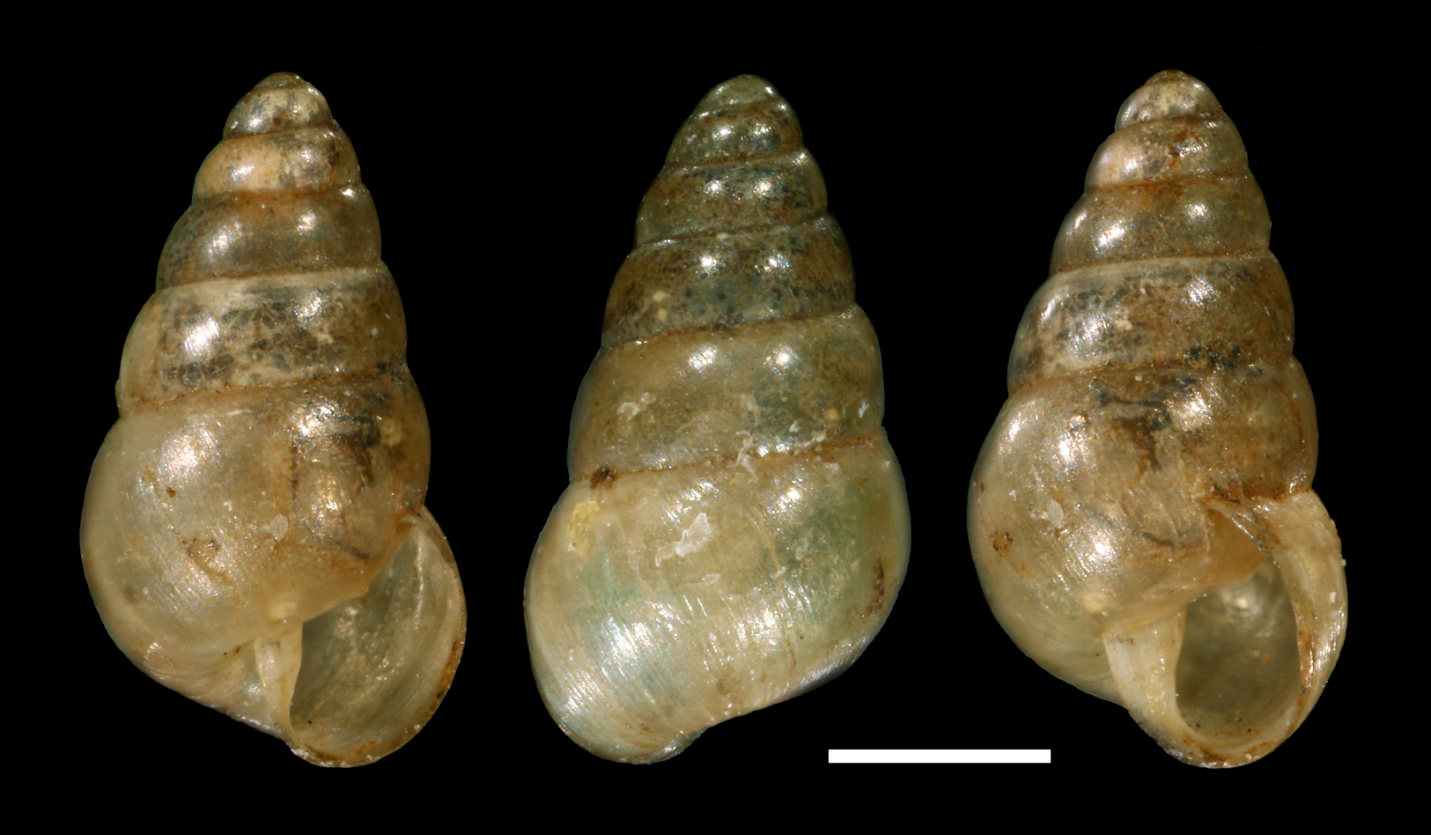
*Tornatellina boeningi* Schmacker & Boettger, 1891 (now *Tornatellides boeningi*), lectotype, SMF 42552, 3.3 mm height, Tamsui, Taiwan; scale bar = 1 mm (Photo credit: Sigrid Hof).

*T. boeningi* was described from Taiwan and Yen (1939) reported additional specimens from Hongkong, and with *T. [b.] inexpectatus* regarded as subspecies or even synonym it is known also from the adjacent Ryukyu Islands and the Izu Islands south of Honshu. Furthermore, it occurs on the Ogasawara Islands as reported by Wada, Kawakami & Chiba (2012) who observed its survival of the passage through a bird’s digestive system thus suggesting a bird-mediated dispersal mode. Conchological comparison to images of the lectotype (Yen, 1939: fig. 26 and Fig. 2) and a Japanese specimen figured by Sasaki (2008: fig. 15) shows great resemblance to our species; the original description gives dimensions of 3.25 mm height and 1.88 mm diameter which falls well within the range of the material from the greenhouse.

Given the choice with no clear conchological preference to either of the two candidate species *T. oblongus* and *T. boeningi*, we tentatively identify our material as *T*. cf. *boeningi* accounting for probabilities: An origin in southeast Asia seems much more likely than in very remote Oceania.

Regarding the type material of *T. boeningi* there is some confusion that should be clarified. Our conclusion is that Yen (1939) correctly designated the lectotype from the original material and that the type locality is Tamsui: While the species was explicitly described from material originating from northern Taiwan [Formosa] (Tamsui) (other than most material dealt with by Schmacker & Boettger (1891) in the same paper which came from Takao, southern Taiwan), Yen (1939) gave as type locality “Takao, Formosa”, both under the species account as well as in the figure caption of the lectotype. A request at the SMF confirmed that the original label by Boettger states “Takao, S. Formosa”, but Zilch as curator at the time added a label with “N-Formosa: Tamsui”. The SMF material originates from collection O. Boettger, ex Schmacker, so the other potential error that Yen selected from non-type material is ruled out. The copy of Yen in the SMF library is annotated by Zilch stating that Boettger’s label is surely erroneous because the species was only mentioned from Tamsui. Thus the lectotype selection by Yen (1939) was based on the correct, but obviously erroneously labelled material—not unlikely given the overall assemblage of material.

#### Molecular identification

Being aware of the remaining uncertainty in the conchological identification of the *Tornatellides* species we tested molecular identification as well.

For two specimens we obtained a partial COI sequence of 658 base pairs (bp), both representing the same haplotype. For 16S we generated a single partial sequence of 420 bp (Table 1).

Searches in GenBank taxonomy and by the BLAST^®^ tool resulted in a total of six partial COI-sequences (two *Tornatellides*, three *Elasmias* and one *Tornatellaria* species) leading to an alignment of 654 bp and 122 partial 16S sequences (one Hawaiian *Tornatellides* and otherwise 37 different haplotypes of *T. boeningi*) aligned to a 417 bp dataset including gaps. For geographic origin and GenBank-accession-numbers see Table 1.

This extremely limited number of available sequences, however including one “target” species, partially with missing species identifications, did not allow meaningful further analyses other than calculating pairwise *p*-distances as a first test of identity.

For neither COI nor 16S sequences did the comparison result in a match. Minimum *p*-distance in COI was 5.4% to an unidentified *Tornatellides* species from Molokai, but even an *Elasmias* species from Maui only showed 5.7% difference (Table 2). Minimum *p*-distance for 16S was expectedly lower (i.e. sequences more similar), but surprisingly the difference to the unidentified Hawaiian Islands representative was lower than to our candidate taxon from the conchological analyses when all its haplotypes were treated as a group (Table 3). However, the 37 *Tornatellides boeningi* haplotypes showed a considerable within-group divergence of 1.1% and in a pair-wise comparison of all different haplotypes, two Hahajima-haplotypes showed less divergence (1.68% and 1.69% respectively) than the Hawaiian specimen and other Hahajima-haplotypes.

**Table 2:** Genetic *p*-distance of the *Tornatellides* species from Gothenburg to the available sequences of related species, partial COI-sequences of 654 base pairs length, for material see Table 1.

Specimens	*p*-distance to *Tornatellides* cf. *boeningi*, Gothenburg
*Tornatellides* sp., Molokai (MT519859)	0.054
*Elasmias* sp., Maui (AY148499)	0.057
*Tornatellides* sp., Oahu (AY148504)	0.063
*Tornatellaria* sp., Maui (MT519858)	0.089
*Elasmias luakahaense*, Oahu (AY148498)	0.153
*Elasmias peaseanum*, Moorea (AY148503)	0.165

**Table 3:** Estimate of genetic *p*-distance between groups: *Tornatellides* species from Gothenburg to other species, partial 16S-sequences with 417 positions in the final dataset, for material see Table 1.

Species	*p*-distance to *Tornatellides* cf. *boeningi*, Gothenburg
*Tornatellides* sp., Molokai (MT519878)	0.019
*Tornatellides boeningi*, Hahajima (37 different haplotypes)	0.023

The interpretation of the “*Tornatellides boeningi*” sequences from Hahajima is further complicated by the fact, that for the Ogasawara Islands, Pilsbry & Cooke (1914–1916) described the endemic species *T. tryoni* (see also above, conchologically ruled out). So far only Wada & Chiba (2011)—based on unpublished COI and 16S sequence data—speculate that “*Tornatellides tryoni* of Ogasawara is phylogenetically closest to *Tornatellides boeningi*, a seashore species of Japan and Ryukyu Islands, and they are most likely identical species”. The identification as “*boeningi*” for the published sequences (Wada, Kawakami & Chiba, 2012) is obviously based on that side remark in Wada & Chiba (2011). Since the type locality of *T. boeningi* is located on northern Taiwan, the potential synonymy of the two species still remains to be proven.

In conclusion, available sequence data neither enable a meaningful identification nor even help to narrow down a potential source area. This is mainly due to the nearly complete absence of accessible comparative data so urgently needed especially in the identification of introduced species.

#### Habitat in the Botanical Garden

**Figure 3:**
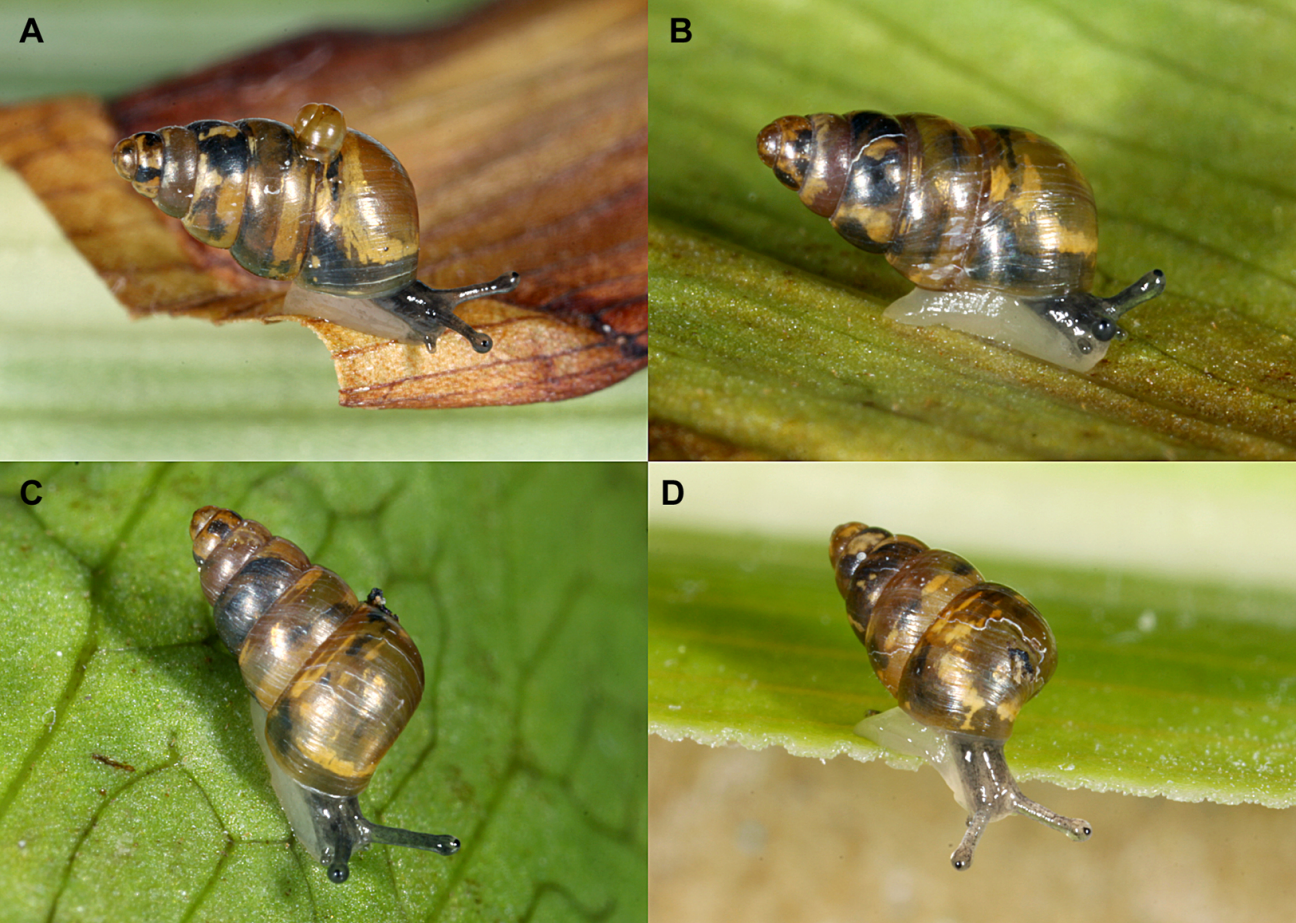
(A–D) Body coloration of four different individuals of *Tornatellides* cf. *boeningi* found in the Gothenburg Botanical Garden, SMNS-ZI0144091 (Photo credit: Ira Richling).

The population of *Tornatellides* cf. *boeningi* in the Gothenburg Botanical Garden concentrates in the Tropical house (highest population density), but also lives in the adjacent orchid houses. Almost all specimens were arboreal, aestivating or crawling on the underside of leaves of several tropical plant species or on petioles and stems (Fig. 3). Others, especially in the orchid houses, were climbing on the outside of the orchid pots. The latter microhabitat was shared by *Afropunctum seminium* (Morelet, 1873), which was first recorded there in 2000 (Proschwitz, 2001, 2004) and still has a viable population, and *Discostrobilops hubbardi* in extremely low density. With its presence in the different houses, *Tornatellides* cf. *boeningi* seems to tolerate minimum temperatures down to 12 °C (and lower humidity), but its highest numbers in the Tropical and *Vanilla*-house suggest a preference for true tropical moist and hot conditions.

### *Discostrobilops hubbardi* (A. D. *Brown, 1861*)

Material investigated: Gothenburg Botanical Garden, *Vanilla*-house: leg. Ira Richling, 18.8.2017 (SMNS-ZI0144093/2 spms.); leg. Ted von Proschwitz, 15.2.2019 (GNM 308506/1 spm.)

**Figure 4:**
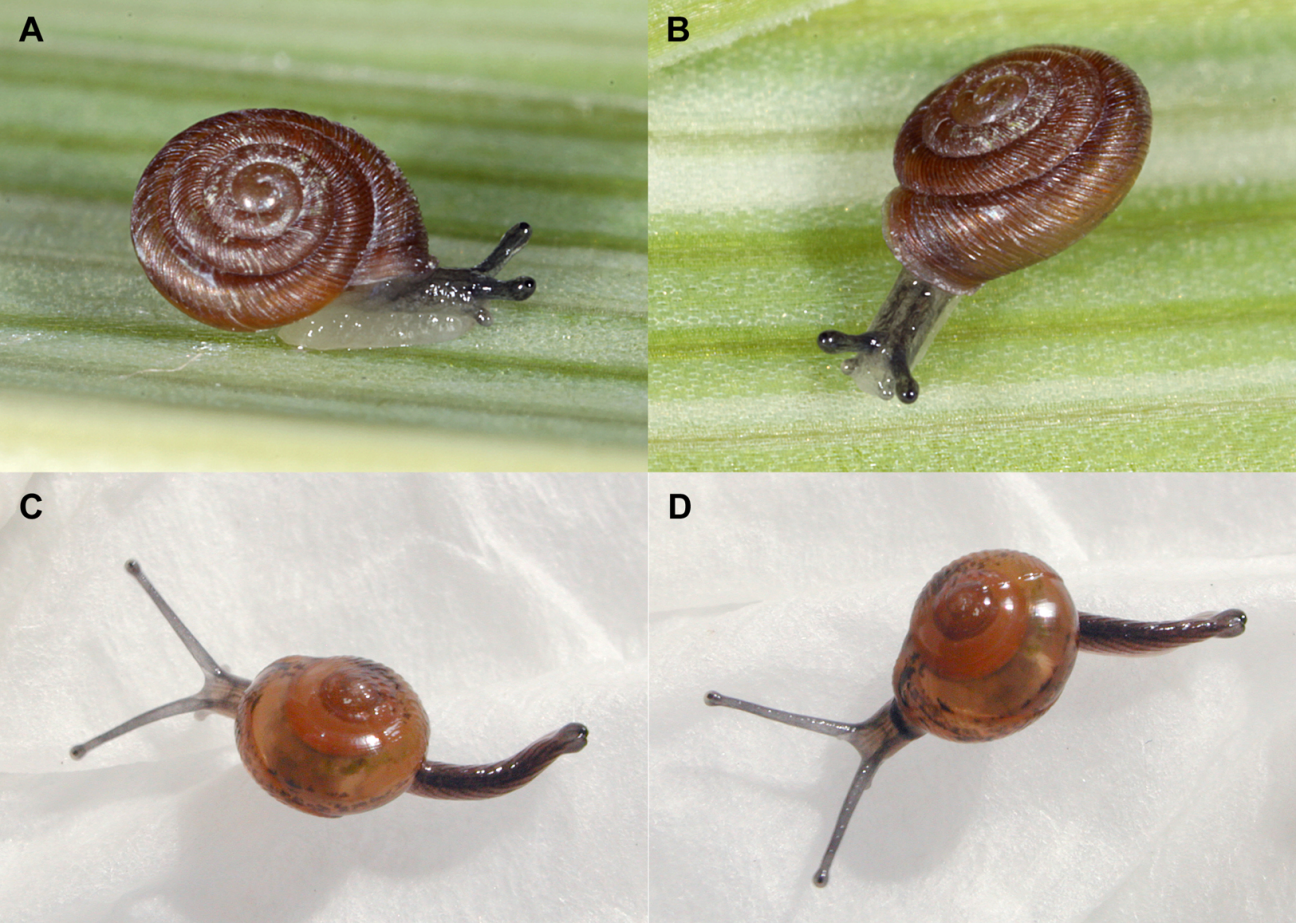
Living snails. (A and B) *Discostrobilops hubbardi* found in the Gothenburg Botanical Garden, SMNS-ZI0144093, about 2.5 mm shell diameter (Photo credit: Ira Richling); (C and D) *Ovachlamys fulgens* found in the Tropical Rainforest house of the Public Science Center Universeum in Gothenburg, GNM 297160, about 4 mm shell diameter (Photo credit: Per Lekholm).

For Europe the species was first spotted and reported in several greenhouses of the Hortus Botanicus in Vienna by Reischütz et al. (2018). These authors discuss the species identification in detail. In Vienna it occurs mostly in houses focused on orchid culture which agrees well with the first specimen detected in the *Vanilla*-house of the Gothenburg Botanical Garden crawling on leaves of hanging orchids (Figs. 4A and 4B).

*Discostrobilops hubbardi* originates from subtropical and tropical areas of the New World, specifically from the coastal plain of the Gulf of Mexico from Florida and Georgia to north-eastern Mexico, Jamaica, Cuba, Bimini islands and Bermuda (Thompson, 2011). The repeated occurrence in greenhouses in combination with orchids suggests that it may be spread with the orchid trade. This is further confirmed by a sighting of this species on an orchid plant in a private house in Stuttgart (no voucher) crawling on the substrate. This orchid, a *Cattleya* species, was bought from a specialized orchid trader, but has since been repotted.

### *Ovachlamys fulgens* (*Gude, 1900*) (Figs. 4C and 4D).

Material investigated: Tropical Rainforest house of the Public Science Center Universeum in Gothenburg (57°41′44.18″ N 11°59′20.67″ E): leg. Ted von Proschwitz, 19.4.2004 (GNM 297160/5 spms. (4 living, 1 empty shell)).

At the time of the discovery the Tropical Rainforest house still harboured a fauna rich in introduced mollusc species such as *Leptinaria lamellata* (*Potiez & Michaud, 1838*) (= *L. unilamellata* (*d’Orbigny, 1838*) according to Breure, Ablett & Audibert (2020)) , *Allopeas clavulinum* (*Potiez & Michaud, 1838*), *Subulina octona* (*Bruguière, 1789*), *Afropunctum seminium*, *Hawaiia minuscula* (*Binney, 1840*), *Zonitoides arboreus* (*Say, 1817*) and *Ambigolimax valentianus* (*Férussac, 1822*) (Proschwitz, 2005, 2016, 2017). In early 2016 the rainforest house was thoroughly converted and during a search in March 2017, neither *L. unilamellata* nor the—at this time still unidentified—species of semi-slug could be re-found, possibly both are now eradicated (cf. Proschwitz, 2017).

*Ovachlamys fulgens* is well known as “travelling around the world”. It was originally described from Naha on the Loo Choo Islands, the old English name for the Ryukyu Islands. Naha is located on Okinawa.

Nowadays it is recorded as introduced and pest species from several places around the world. Records arranged by time of first recognition are known from the following places: Costa Rica (about early 1980’s, Barrientos, 1998 and IR personal observation since 1997), American Samoa—Tutuila and Olosega (1998, Cowie, 2000, 2001; Cowie et al., 2002), Hawaiian Islands: O’ahu and Hawai’i (1999, Cowie, 2000), Kaua’i and Maui (2005, 2004, Hayes, Tran & Cowie, 2007), USA, Florida (2001, Robinson & Slapcinsky, 2005), Trinidad (2004, Robinson & Slapcinsky, 2005), Tobago (2012, Rutherford & Mohammed, 2013), Brazil (2015, Teixeira, Cunha & Bornschein, 2017). Furthermore, it was intercepted in shipments from Central America, (SE Asia) and Oceania (Robinson, 1999) as well as from Thailand, Singapore, Colombia, Trinidad & Tobago (Robinson & Slapcinsky, 2005). In an overall assessment of introduced species from a United States perspective, Cowie et al. (2009) attributed a fairly high pest potential to *Ovachlamys fulgens* (14 on a scale from 1 to 46 with 1 being highest).

A previous record from a greenhouse is known from Chicago (Robinson & Slapcinsky, 2005), however, the record from the Public Science Center Universeum in Gothenburg is the first reported case from a greenhouse in Europe.

## Discussion

### Horticultural trade

Leonhardt & Sewake (1999), in a guide on *Dendrobium* orchid culture in Hawaii, list a native unidentified *Tornatellides* species as one of the seven significant molluscan pest species in these orchid cultures. No damage caused by these tiny snails is indicated but they are noted as significant for quarantine in the ornamental plant trade. The species figured by Leonhardt & Sewake (1999: fig. 3.34) looks very similar to the species occurring in the Gothenburg Botanical Garden with respect to general shell shape and also animal coloration (Fig. 3). However, there are too many and possible additional undescribed native tornatellidine species recorded from the Hawaiian Islands of roughly similar appearance and the image does not show all diagnostic characters to conclude identity of the species. Cowie et al. (2008: 270) also mention several presumably native species of unidentified Tornatellidinae (“probably all *Tornatellides* spp. but possibly including some *Tornatellaria* spp.”) in their survey of horticultural facilities on the Hawaiian Islands which could hint at multiple sources of *Tornatellides* species in greenhouses. Considering the recent find and the observations by Leonhardt & Sewake (1999), it should be investigated if those occurrences stem from native Hawaiian species as assumed by Cowie et al. (2008). Also *Ovachlamys fulgens* was found in nine of 40 surveyed horticultural facilities on four of the Hawaiian islands, which also suggests the spread with the trade of plants.

Like all *Tornatellides* species, the greenhouse dweller is ovoviviparous (a typical juvenile can be seen on top of an adult specimen in Fig. 3) and thus giving it one important feature shared by many successful introduced molluscan species, e.g. *Afropunctum seminium* and other euconulid species. Furthermore, its small size combined with the mostly arboreal lifestyle results in a fairly firm attachment to the substrate, in aestivation as well as the crawling animals, which makes it an excellent traveller on plants.

### Feasibility of molecular identification

As our attempt of molecular identification failed, we checked the potential for other species reported from horticultural facilities (Table 4). For about one third (10) of the 29 species chosen to check for coverage in GenBank, sequence data for the barcoding fragment of COI were missing, the same applies for twelve species regarding 16S sequence data. For another five species, only one or two accessions are available. When looking at the greater context for potential species determination, i.e. sequence data presence of species within the same genus, a similar picture shows up with data for half of the genera totally missing in GenBank or with only one or two species each. In the latter cases, often just the species reported from horticulture is present, impressive examples being *Lissachatina*, *Veronicella* and *Parmarion*. So, even if a species is represented by COI sequence data, the scarcity of accessions or missing comparative sequences from related species illustrating the potential range of haplotype variation may hamper a possible genetic identification attempt.

**Table 4:** Number of sequences available on GenBank for selected non-operculate land snail species for the mitochondrial cytochrome oxidase subunit 1 (COI) (in parenthesis with less than 500 base pairs), and the mitochondrial 16S ribosomal RNA gene.

Species	Family	COI	16S	Comparative data on generic level
*Deroceras laeve* (*Müller, 1774*)	Agriolimacidae	184 (179)	15	6/104
*Lissachatina fulica* (*Bowdich, 1822*)	Achatinidae	115 (17)	33	1/16
*Cornu aspersum* (*Müller, 1774*)	Helicidae	58	458	1/1
*Zonitoides arboreus* (*Say, 1817*)	Gastrodontidae	53	–	2/16
*Euglandina rosea* (in *Ferussac 1921–1822*, see also under *Ambigolimax valentianus*)	Spiraxidae	52	43	3/52
*Sarasinula plebeia* (*Fischer, 1868*)	Veronicellidae	44	87	2/3
*Bradybaena similaris* (in *Ferussac 1921-1822*, see also under *Ambigolimax valentianus*)	Camaenidae	12 (11)	15	7/60
*Ovachlamys fulgens* (*Gude, 1900*)	Helicarionidae	8	1	1/2
*Subulina octona* (*Bruguière, 1789*)	Achatinidae	7	2	3/56
*Eleutherocaulis alte* (in *Ferussac 1921–1822*, see also under *Ambigolimax valentianus*) (= *Laevicaulis alte*)	Veronicellidae	6 (4)	4	3/9
*Gulella bicolor* (*Hutton, 1834*)	Streptaxidae	4	1	36/475
*Veronicella cubensis* (*Pfeiffer, 1840*)	Veronicellidae	4 (3)	6	1/5
*Gastrocopta servilis* (*Gould, 1843*)	Vertiginidae	3	2	22/91
*Polygyra cereolus* (*Megerle von Mühlfeldt, 1818*)	Polygyridae	3 (2)	4	2/9
*Tayloria quadrilateralis* (*Preston, 1910*) (= *Gonaxis quadrilateralis*)	Streptaxidae	2	1	3/21
*Paropeas achatinaceum* (*Pfeiffer, 1846*)	Achatinidae	2	–	1/4
*Allopeas clavulinum* (*Potiez & Michaud, 1838*)	Achatinidae	2	1	2/17
*Parmarion martensi* *Simroth, 1893*	Ariophantidae	1	–	1/9
*Meghimatium striatum* *Van Hasselt, 1824*	Philomycidae	1	1	7/10
*Tayloria kibweziensis* (*Smith, 1894*) (= *Gonaxis kibweziensis*)	Streptaxidae	–	1	3/21
*Succinea tenuis* Morelet, 1865 (= *Succinea tenella* Morelet, 1875)	Succineidae	–	–	14/150
*Succinea costaricana* in Martens (1890–1901)	Succineidae	–	–	14/150
*Allopeas clavulinum kyotoense* (*Pilsbry & Hirase, 1904*) (= *Allopeas kyotoense*)	Achatinidae	–	–	2/17
*Lamellidea oblonga* (*Pease, 1865*)	Achatinellidae	–	–	0/17
*Kaliella doliolum* (*Pfeiffer, 1846a*) (= *Liardetia doliolum*)	Chronidae/ Euconulidae	–	–	0/115 *Liardetia*: 1/33
*Beckianum beckianum* (*Pfeiffer, 1846b*)	Achatinidae	–	–	0/3
*Opeas hannense* (*Rang, 1831*)	Achatinidae	–	–	0/67
*Hawaiia minuscula* (*Binney, 1841*)	Pristilomatidae	–	–	0/5
*Discostrobilops hubbardi* (*Brown, 1861*)	Strobilopsidae	–	–	0/2 *Strobilops*: 1/11

**Note:**

List based on species recorded in horticultural facilities on the Hawaiian Islands according to Cowie et al. (2008), Hawaiian native species excluded, and *Discostrobilops hubbardi* from this study; nomenclature updated according to MolluscaBase (2020). Comparative data = number of species of the genus with sequence for COI in GenBank/number of accepted species in genus according to MolluscaBase (2020).

Since the test of the first necessary step towards an identification, i.e. the availability of any sequence information, already largely failed, we regarded any further analyses of the available data quality, especially with respect to the proper identification, as meaningless at this stage. Obviously, a sequence match with available data would only mean a similarity with the respective voucher specimen, which, however, would at least provide the important information about geographic origin (if provided along with the sequence data), but clearly not guarantee a correct identification. This was exemplified in the discussion of the *Tornatellides boeningi*-sequences provided by Wada, Kawakami & Chiba (2012) whose material may even stem from a different species. In any way, their data and the majority of all other sequences checked here, are not based on material from the type locality (or type material) or not even from the general area of the species’ origin. This would clearly be the basic requirement for a true DNA reference library for correct identification, with all known additional problems at the level of the genetic data itself not discussed here.

In conclusion, any reports on introduced species are strongly encouraged to provide genetic reference data as well if facilities and funding are available. While species from temperate regions are better covered (e.g. Blacket et al., 2016; and see Table 4: *Deroceras laeve, Cornu aspersum, Zonitoides arboreus*), this concerns especially tropical species where resources in the source countries may be very limited. A good example is provided by Gittenberger, Reijnen & Groenenberg (2019) in a study on the Maldives and these authors also point out the scarcity of basic molecular information.

## Supplemental Information

10.7717/peerj.11185/supp-1 Supplemental Information 1 New COI sequence of Tornatellides cf. boeningi.

10.7717/peerj.11185/supp-2 Supplemental Information 2 New 16S sequence of Tornatellides cf. boeningi.

## Abbreviations

GNM: Gothenburg Natural History Museum, Sweden
SMF: Forschungsinstitut und Natur-Museum Senckenberg, Frankfurt, Germany
SMNS: Staatliches Museum für Naturkunde Stuttgart, Germany (Stuttgart State Museum of Natural History)
spm./spms.: specimen/specimens

## Additional Information and Declarations

### Data Availability

The following information was supplied regarding data availability: New sequences are available at GenBank: MW354671 and MW354514.
